# Drug-induced mild therapeutic hypothermia obtained by administration of a transient receptor potential vanilloid type 1 agonist

**DOI:** 10.1186/1471-2261-10-51

**Published:** 2010-10-09

**Authors:** Keld Fosgerau, Uno J Weber, Jacob W Gotfredsen, Magdalena Jayatissa, Carsten Buus, Niels B Kristensen, Mogens Vestergaard, Peter Teschendorf, Andreas Schneider, Philip Hansen, Jakob Raunsø, Lars Køber, Christian Torp-Pedersen, Charlotte Videbaek

**Affiliations:** 1 Neurokey AS, Diplomvej 372, DK-2800 Lyngby, Denmark; 2 Pipeline Biotech A/S, Røvedvej 1, Spørring, DK-8380 Trige, Denmark; 3 Department of Animal Health and Bioscience, Faculty of Agricultural Sciences, Aarhus University, Box 50, DK-8830 Tjele, Denmark; 4 Department of Anesthesiology and Intensive Care Medicine, University Hospital of Cologne, Kerpener Strasse 62, D-50937 Cologne, Germany; 5 CMC Consult Aps, Agern Allé 3, DK-2970 Hørsholm, Denmark

## Abstract

**Background:**

The use of mechanical/physical devices for applying mild therapeutic hypothermia is the only proven neuroprotective treatment for survivors of out of hospital cardiac arrest. However, this type of therapy is cumbersome and associated with several side-effects. We investigated the feasibility of using a transient receptor potential vanilloid type 1 (TRPV1) agonist for obtaining drug-induced sustainable mild hypothermia.

**Methods:**

First, we screened a heterogeneous group of TRPV1 agonists and secondly we tested the hypothermic properties of a selected candidate by dose-response studies. Finally we tested the hypothermic properties in a large animal. The screening was in conscious rats, the dose-response experiments in conscious rats and in cynomologus monkeys, and the finally we tested the hypothermic properties in conscious young cattle (calves with a body weight as an adult human). The investigated TRPV1 agonists were administered by continuous intravenous infusion.

**Results:**

Screening: Dihydrocapsaicin (DHC), a component of chili pepper, displayed a desirable hypothermic profile with regards to the duration, depth and control in conscious rats. Dose-response experiments: In both rats and cynomologus monkeys DHC caused a dose-dependent and immediate decrease in body temperature. Thus in rats, infusion of DHC at doses of 0.125, 0.25, 0.50, and 0.75 mg/kg/h caused a maximal ΔT (°C) as compared to vehicle control of -0.9, -1.5, -2.0, and -4.2 within approximately 1 hour until the 6 hour infusion was stopped. Finally, in calves the intravenous infusion of DHC was able to maintain mild hypothermia with ΔT > -3°C for more than 12 hours.

**Conclusions:**

Our data support the hypothesis that infusion of dihydrocapsaicin is a candidate for testing as a primary or adjunct method of inducing and maintaining therapeutic hypothermia.

## Background

Mild therapeutic hypothermia (target temperature between 32°C and 34°C) has emerged as an effective treatment to improve neurological outcomes among cardiac arrest survivors. Following publication of two pivotal randomized clinical trials [1-3] mild therapeutic hypothermia for 12 to 24 hours following cardiopulmonary resuscitation (CPR) of out of hospital cardiac arrest patients with shockable electrocardiography rhythms is recommended by the European Resuscitation Counsel (ERC) and the American Heart Association (AHA) for prevention of neurological injury. Also, mild therapeutic hypothermia has been suggested to improve the outcome in other hypoxic-ischemic conditions including acute ischemic stroke [4], acute myocardial infarction [5], and neonatal encephalopathy [6]. At present therapeutic hypothermia of human subjects is obtained with a variety of methods, which combine physical cooling of the body surface or blood stream with anesthesia and relaxation to inhibit shivering. In general these physical/mechanical methods are cumbersome and associated with side-effects [7-10]. Therefore cooling of the body by changing the temperature homeostasis with a drug would offer an attractive alternative to the existing treatment, since it potentially would be easy to manage and the need for anesthesia and relaxation may be avoided.

Temperature regulation is a complex biological process controlled by the pre-optic area of the anterior hypothalamus as well as peripheral thermo-sensors [11]. Recently it has been demonstrated that ion channels of the transient receptor potential (TRP) family are pivotal in this regulation and involved in the peripheral mechanisms by which we sense hot and cold temperatures [11,12]. A central member of the TRP family is the transient receptor potential vanilloid type 1 (TRPV1), formerly known as the capsaicin-vanilloid receptor-1, which was cloned in 1997 [13]. The TRPV1 receptor is widely expressed in the human body particularly in "port-of-entry" tissues, the peripheral nervous system, and in the brain [14,15]. Due to the involvement of TRPV1 in nociception it has been widely studied as a target for the development of drugs [16-18] especially for the treatment of pain [19].

The TRPV1 receptor is a target for a large heterogeneous group of natural compounds including capsaicinoids such as capsaicin and dihydrocapsaicin (DHC) from chili pepper, piperine from black pepper, resiniferatoxin (RTX), ginsenosides, evodiamine and others that acts as agonists [20]. In addition potent synthetic agonists have been developed including rinvanil [21], MSK-195 [22], arvanil [23], and olvanil [24]. Interestingly, administration of TRPV1 agonists has been demonstrated to induce hypothermia in rats [25,26]. However, it is currently not known whether this hypothermia induced by infusion of a TRPV1 agonist can be maintained at a clinically relevant duration, or whether the effect is restricted to rodents or can be observed in large animals or humans with a lower body surface. Importantly, agonists of TRPV1 have been shown to display differential effects concerning receptor desensitization [27], which may affect the sustainability of the hypothermic properties.

The objective of the present study was to evaluate the feasibility of pharmacologically induced hypothermia in several species *in vivo*. In particular we hypothesized that infusion of a TRPV1 agonist may control body temperature in a fashion, which holds the potential for obtaining drug induced mild therapeutic hypothermia in survivors after out of hospital cardiac arrest.

## Methods

### Rat hypothermia screen model

#### Animals

Hypothermia screening studies of TRPV1 agonists were performed at Pipeline Biotech AS (Trige, Denmark) and initiated in Sprague Dawley male rats (Taconic Europe, Lille Skensved, Denmark) aged 7-9 weeks weighing approximately 250 grams. Animals were kept in a temperature-controlled environment (20-24°C) at a 12 hour normal dark-light cycle (lights on at 06.00 a.m.) with free access to chow (Altromin 1324, Altromin Spezialfutter GmbH, Lage, Germany) and UV-sterilized water. The study was conducted in accordance with internationally accepted guidelines for the care and use of laboratory animals and approved by the Danish Committee for Animal Research.

#### Surgery and infusion

Prior to the infusion, the rats were set with a jugular vein catheter and an intraperitoneal (i.p.) chip for temperature measurement. Briefly, a catheter was set in the right jugular vein under sterile conditions in ketamine/xylazine (Intervet Int. B.V., Boxmeer, The Netherlands) anesthetized rats, and the catheter was externalized through the neck to allow free movement during the time of the infusion. Also, for monitoring of i.p. body temperature, a chip sensor (IPTT-300, Electronic ID Transponders, PLEXX, Elst, the Netherlands) was inserted in the peritoneal cavity of the rats. Following the surgery the rats were allowed to recover for 1-3 days before the start of the experiment. Then, on the day of the infusion the rats were treated with carprofen (5 mg/kg, Rimadyl vet.^®^, Pfizer Inc., NY, USA), placed in a Culex Raturn movement responsive cage (BASi, West Lafayette, IN, USA) and connected to the peristaltic pump (Gilson, Villiers le Bel, France). The rats were allowed to recover for 1 hour before body temperature was recorded every 15 minutes starting at t = -15 minutes (baseline) and until t = 6 hours using the wireless reader (BMDS DAS-6007, IPTT Data Acquisition System, PLEXX, Elst, the Netherlands). Rats with an initial temperature reading below 36°C as a result of a misplaced chip were excluded from the experiment. The infusion of test compounds was started at t = 0 hours and continued for 4 hours. Rats (n = 2-6 per group) were administered the following compounds infused at a constant flow-rate of 2 ml/kg/h: Vehicle control (2% polysorbate 80 (Sigma Aldrich, St. Louis, MO, USA) in saline); Dihydrocapsaicin, 0.6 mg/kg/h (Cat. 92355, Cayman Chemical Company, AH Diagnostics, Aarhus, Denmark); Resiniferatoxin, 0.06 mg/kg/h (R8756, Lot. 038K1583, Sigma Aldrich, St. Louis, MO, USA); Olvanil, 3.0 mg/kg/h (A2098, Lot. 122K4620, Sigma Aldrich, St. Louis, MO, USA); Arvanil, 3.0 mg/kg/h (A2098, Lot. 122K3420, Sigma Aldrich, St. Louis, MO, USA); Rinvanil, 15 mg/kg/h (Giovanni Appendino, University Del Piemonte Orientale, Italy); MSK-195, 2 mg/kg/h (Cat. ALX-550-396-M005, L13899, Medinova Scientific A/S, Glostrup, Denmark). Following the infusion the animals were euthanized by CO_2_.

### Dose-response of dihydrocapsaicin in rat

#### Animals

Dose-response experiments of DHC were performed at University of Heidelberg, Department of Anesthesiology (Heidelberg, Germany) and initiated in male Wistar-Han rats (n = 30, Elevage Janvier, Le Genest St., Ille, France) weighing 300-350 grams. Animals were kept at a humidity of 55 ± 5% in a temperature-controlled environment (20 ± 1°C) and at a 12 hour normal dark-light cycle (lights on at 07.00 a.m.) with free access to chow (Ssniff RM/H, Ssniff Spezialdiäten GmbH, Soest, Germany) and tap water unless otherwise stated. All experiments were conducted in accordance with European Communities Council Directive of 24^th ^November 1986 (86/609/EEC) and approved by the German governmental Animal Care Committee.

#### Surgery and infusion

The animals were implanted with PE-50 infusion catheter in the left femoral vein under pentobarbital anesthesia. The catheter was exposed at the back of the animal through a tunnel and secured by a Covance infusion harness (Instech Laboratories, Plymouth Meeting, PA, USA). Also, temperature probes (VitalView Series 4000 G2 E-Mitter, Mini Mitter Company, Bend, OR, USA) were implanted in the peritoneal cavity. Following 24 hours of recovery the rats were treated with carprofen (5 mg/kg i.v., Rimadyl^®^, Pfizer Pharma GmbH, Karlsruhe, Germany) as analgesic treatment, connected to the infusion pump (Compact infusion pump 975, Harvard Apparatus, Holliston, MA, USA) and stratified into 5 groups (n = 6 per group) based on post-surgery body weight. Following a 1 hour recovery period body temperature was recorded telemetrically using the VitalView system. The temperature was recorded before (t = -1 to 0 hours), during (t = 0 to 6 hours) and after (t = 6 to 10 hours) the infusion of vehicle control (2% polysorbate 80 (Fluka Chemie AG, Buchs, Switzerland) in saline) or DHC (Cat. 92355, Cayman Chemical Company, AH Diagnostics, Aarhus, Denmark). Solutions were infused continuously via the femoral catheter at an infusion rate of 0.013 ml/h in all animals with doses of DHC at 0, 0.125, 0.25, 0.50 or 0.75 mg/kg/h.

### Dose-response of dihydrocapsaicin in cynomologus monkeys

#### Animals

Two adult cynomologus monkey (Macaca fascicularis) with a body weight of 3 kg (female) and 6 kg (male), respectively, were purchased at Conghua Farm, Quigan Town, China and Nafovanny, Long Thanh, Vietnam. Animals were single housed at Covance GmbH facilities (Münster, Germany) in a normal 12 hour light cycle (lights on at 05.30 a.m.) in a climate controlled room with >10 air changes/h and temperature and relative humidity ranges of 19 - 25°C and 40 - 70%, respectively. Twice daily the animals were offered a commercial pellet diet for primates (Ssniff P10, Ssniff Spezialdiäten GmbH, Soest, Germany), and in addition the animals received fresh fruit and bread as food supplement. Tap water was provided *ad libitum*. The study was conducted in accordance with European Communities Council Directive of 24^th ^November 1986 (86/609/EEC) and International Conference on Harmonization (ICH) guidelines and approved by the German governmental Animal Care Committee.

#### Surgery and infusion

The animals were anesthetized with ketamine (Ketavet^®^, Pfizer Pharmacia & Upjohn GmbH, Berlin, Germany), atropine (Eifelfango, Bad Neuenahr, Germany) and xylaxine (Rompun^® ^2%, Bayer Vital GmbH, Leverkusen, Germany) for the implantation of the catheter and the port system (Port-a-cath Implantable Access System, SIMS Deltec Inc., St. Paul, MN, USA). Briefly, the port was implanted subcutaneously in the middle of the back and a catheter was tunneled under the skin via the femoral vein into vena cava caudalis for intravenous infusion. Also, the animals were implanted with a temperature chip (IPTT-300 transponders, PLEXX, Elst, the Netherlands) in the intraperitoneal cavity for measurement of body temperature. Following the surgery the animals were given analgesic treatment and allowed to recover for 2-3 weeks. During the recovery period catheters were flushed regularly with normal saline to maintain the free flow of the port catheter system, and the monkeys were allowed to adapt to the individually fitted jacket, which covered the area above the port and stored the infusion pump. Following recovery the conscious and freely moving cynomologus monkeys were subjected to a 12 hour infusion of vehicle control or DHC using a Pegasus Pump Vario (Uno Roestvastaal BV, PX Zevenaar, the Netherlands) fitted in a backpack system. Studies were performed with at least three days wash-out period between the study days, and starting with the vehicle control followed by doses of DHC of 0.3, 0.6., 1.2, and 1.8 mg/kg/h. The infusions were step-wised increased into the full flow-rate such that flow-rate was at 25% from t = 0 to 30 minutes, then 50% from t = 30 to 60 minutes, and 100% from t = 1 to 12 hours. Dihydrocapsaicin (Lot. 63908, Clauson Kaas AS, Farum, Denmark) was formulated as an emulsion using 0.6% polysorbate 80 (Fluka, Seelze, Germany) and 0.6% MCT oil (Crodamol GTCC, Croda Inc., Snaith Goole, UK) per 3 mg/ml of DHC in 1.6% of glycerol. The vehicle control was 0.6% polysorbate 80 in 1.6% of glycerol. Temperature readings were performed using a DAS-6003 Mini Power System and DASWR-6007 reader (PLEXX, Elst, the Netherlands) at t = 0, 15, 30, 45, 60, 120, 180, 240, 300, 360, 420, 480, 540, 600, 660, 720, 735, 750, 765, 780, 840, 900, 960 minutes.

### Long-tem infusion of dihydrocapsaicin in young cattle

#### Animals

The calf experiments were carried out at the intensive care facility at the Faculty of Agricultural Sciences, Aarhus University, Denmark. Danish Holstein (Bos taurus) calves weighing 71-86 kg were fed 4.5 kg milk-replacer in the morning (123 g DM/kg, Friska Sød, DLG, Copenhagen, Denmark) and starter concentrate (maximal allowance 1000 g as fed/d, ValseStart Grøn, Brødrene Ewers A/S, Sønderborg, Denmark). Dry feed was removed from calves at 12.00 a.m. the day before surgery and calves were not fed milk replacer until after surgery. Calves were kept under a 16/8 hour light/dark program and the rooms were heated and equipped with mechanical ventilation. The temperature was set to 20°C and the calves were loose housed individually and tied for one hour per day at time of milk-feeding to get accustomed to the experimental sampling situation. The experimental procedures were conducted under protocols approved by the Danish Animal Experiments Inspectorate (license 2007/561-1381; Niels B. Kristensen) and complied with the Danish Ministry of Justice Law no 382 (June 10, 1987), Act no. 726 (September 9, 1993) concerning animal experimentation and care of experimental animals.

#### Surgery and infusion

General anesthesia was induced by injection of xylazine (0.15 mg/kg, i.m.) and ketamine (3 mg/kg, i.v.) and maintained with isoflurane (2.5%). Calves were intubated and mechanically ventilated with pure oxygen. Then, an incision was placed medial to the right external jugular vein and a purse string suture (4-0 Dermafil, Kruuse, Denmark) was placed on the right common carotid artery and subsequently a catheter was implanted (Tygon S-54-HL,10-15 cm heparinized tip/90 cm total length, 1.27 mm i.d. × 2.29 mm o.d., Buch & Holm AS, Herlev, Denmark). Also, a purse string suture was placed on the right external jugular vein and catheter was implanted (Tygon S-54-HL, 15-22.5 cm heparinized tip/90 cm total length, 1.27 mm i.d. × 2.29 mm o.d.). Catheters were exteriorized at the level of the right shoulder and protected by a patch mounted with 4 skin sutures (Dermafil USP 2, Kruuse, Denmark). Following surgery the catheters were flushed with saline and blocked with saline containing 100 IU heparin/ml, 0.1% benzyl alcohol, and 0.2% cefuroxime sodium (Zinacef^®^, GlaxoSmithKline, Brøndby, Denmark) and calves were injected with flunixin meglumin (Flunixin vet.^®^, ScanVet Animal Health A/S, Fredensborg, Denmark) for 3 days and antibiotics (Streptocillin vet.^®^, Boehringer Ingelheim Denmark AS, Copenhagen, Denmark) for 5 days.

The day before each experimental sampling calves were moved to the sampling rooms. Following shaving, skin disinfection and injection of 3 ml of lidocaine (Xylocain^® ^20 mg/ml, s.c., AstraZeneca A/S, Albertslund, Denmark) calves were fitted with acute catheters (Tygon, S-54-HL, 1.02 mm i.d., 1.78 mm o.d, Buch & Holm A/S, Herlev, Denmark) inserted 15 cm into the left jugular vein by percutaneous venipuncture using a hypodermic needle (Mediplast AB, 2.5 × 110 mm, Malmö, Sweden). The catheter was secured by skin sutures and by 2 cuffs (5 mm pieces of Tygon pump tubing, Buch & Holm A/S, Herlev, Denmark) slid over the catheters using a pair of hemostats after removal of the hypodermic needle. DHC (Cat. 92355, Cayman Chemical Company, AH Diagnostics, Aarhus, Denmark) was dissolved in 20% polysorbate 80 (Sigma Aldrich, St. Louis, MO, USA) in saline to a concentration of 12 mg/ml. Solutions of DHC (n = 8) or vehicle control (n = 2) were applied as a continuous intravenous infusion using a peristaltic pump (Ole Dich Instrumentmakers Aps, Hvidovre, Denmark). Data recording of temperature via a thermocouple (MLT 1401; AD Instruments, Chalgrove, England) in superior vena cava was initiated 1 hour before (baseline) the start of infusion of DHC and continued for 25 hours. All calves were euthanized at the end of each experimental sampling period.

### Statistical analysis

Data are expressed as mean ± standard error (SE) and compared by a one-way or two-way ANOVA and appropriate post-test, unless otherwise stated. P < 0.05 was considered significant. The difference in body temperature over time is expressed as area under the curve (AUC). All statistical calculations were performed using GraphPad Prism version 4.00 (GraphPad Software, Inc., San Diego, CA).

## Results

### Rat hypothermia screen model

**Figure 1:**
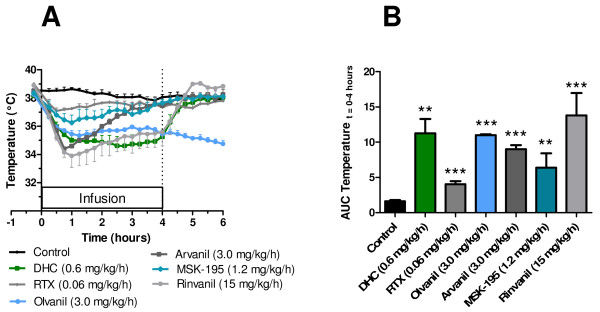
**Effect of TRPV1 agonist on temperature in rat**. (A) Temperature profiles for transient receptor potential vanilloid type 1 agonists dihydrocapsaicin, resiniferatoxin, olvanil, arvanil, MSK-195 and rinvanil, and vehicle control during 4 hour intravenous administration to conscious rats. (B) Area under the curve during the infusion (t = 0 to 4 hours). Statistics: Each of the transient receptor potential vanilloid type 1 agonists were compared to the vehicle control by an unpaired student's t-test ***p < 0.001, **p < 0.01. Values are expressed as average ± SE, with n = 2 - 6, see Methods for further details.

We tested the ability of six TRPV1 agonists including DHC, RTX, rinvanil, olvanil, arvanil and MSK-195 to induce hypothermia in conscious rats by continuous intravenous infusion. All the tested TRPV1 agonists caused a significant decrease of the body temperature during the 4 hours of infusion (Figure 1B, AUC, **p < 0.01), and the maximal response of all the tested agonists as compared to vehicle control was reached within 1 hour of infusion (Figure 1A). Interestingly, the tested agonists differentiated in the degree of tolerance developed towards the hypothermia inducing effect during the infusion. Thus, apparently tolerance development was most pronounced for arvanil with a maximal hypothermia of 34.4°C (t = 0.75 hours) increasing to 37.5°C at the end of the infusion (t = 4 hours). Likewise, rats treated with rinvanil and MSK-195 increased from 33.9 (t = 1.0 hours) to 35.6°C and 36.3 (t = 1.0 hours) to 37.6°C, respectively. The magnitude of the hypothermia effect was dose-dependent for all the tested TRPV1 agonists (see below for DHC). Notably, at the tested doses several of the TRPV1 agonists were able to cause a decrease of body temperature ≥ -3°C (DHC, rinvanil, arvanil, and olvanil). When the infusion was stopped we observed a rapid return to normo-thermia in the DHC treated rats, while a slight hyperthermia was seen in rats treated with rinvanil. In contrast, in rats treated with olvanil we observed a prolonged hypothermia during the entire observation period of 6 hours. Clinical observations included redness of ears and paws, inactivity and flat posture.

### Dose-response of dihydrocapsaicin in rats

**Figure 2:**
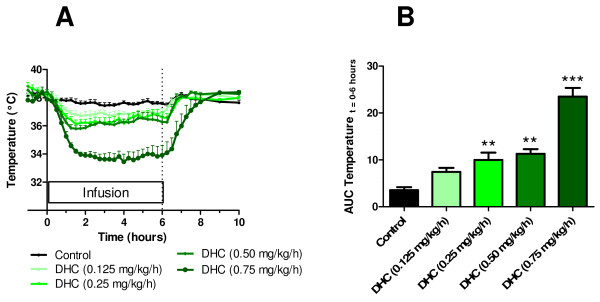
**Hypothermic effects of DHC infusion in rats**. (A) Dose-response temperature profiles in conscious rats during 6 hour intravenous infusion of the transient receptor potential vanilloid type 1 agonist dihydrocapsaicin at doses of 0.125, 0.25, 0.50, and 0.75 mg/kg/h. (B) Area under the curve during the infusion (t = 0 to 6 hours). Statistics: Each of the transient receptor potential vanilloid type 1 agonists were compared to the vehicle control by an unpaired student's t-test ***p < 0.001, **p < 0.01. Values are expressed as average ± SE, with n = 5, see Methods for further details.

Dihydrocapsaicin caused a dose-dependent decrease in body temperature in conscious rats by 6 hour continuous intravenous infusion. As compared to vehicle control the maximal ΔT (°C) observed was -0.9 (t = 1.25 hours), -1.5 (t = 1.5 hours), -2.0 (t = 1.5 hours) and -4.2 (t = 4 hours) at doses of DHC of 0.125, 0.25, 0.50, and 0.75 mg/kg/h, respectively (n = 6/group, Figure 2A). The decrease in body temperature was significant at a dose of DHC of 0.25 mg/kg/h and above (Figure 2B, AUC, **p < 0.01). Notably, when tested at 0.75 mg/kg/h infusion of DHC caused a constant ΔT ≥ -3°C from t = 1.25 to 6.50 hours. Clinical observations included redness of ears and paws, inactivity and flat posture and were dose-dependent i.e. most pronounced at the highest doses tested.

### Dose-response of dihydrocapsaicin in cynomologus monkeys

**Figure 3:**
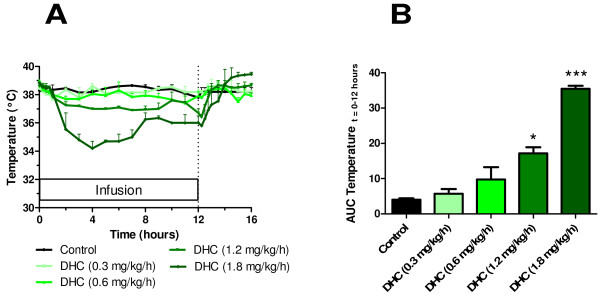
**Hypothermic effects of DHC infusion in monkeys**. (A) Dose-response temperature profiles in conscious cynomologus monkeys during 12 hour repeated intravenous infusions of the transient receptor potential vanilloid type 1 agonist dihydrocapsaicin at doses of 0.3, 0.6, 1.2, and 1.8 mg/kg/h. (B) Area under the curve during the infusion (t = 0 to 12 hours). Statistics: Each of the transient receptor potential vanilloid type 1 agonists were compared to the vehicle control by a one-way ANOVA followed by Dunnett's post-test ***p < 0.001, *p < 0.05. Values are expressed as average ± SE, with n = 2, see Methods for further details.

Repeated administration of DHC by 12 hour continuous intravenous infusion caused a dose-dependent decrease in body temperature in conscious cynomologus monkeys. I.e. as compared to the vehicle control situation the maximal ΔT (°C) observed was -0.7 (t = 1 hours), -0.8 (t = 2 hours), -1.7 (t = 7 hours) and -4.0 (t = 4 hours) at doses of DHC of 0.3, 0.6, 1.2, and 1.8 mg/kg/h, respectively (Figure 3A). The decrease in body temperature was significant at a dose of DHC of 1.2 mg/kg/h and 1.8 mg/kg/h as evaluated by the area under the curve (Figure 3B, *p < 0.05). Notably, we observed development of some tolerance as well as a slight end-infusion hyperthermia when the monkeys were given the highest dose of DHC of 1.8 mg/kg/h. Clinical observations were evident only at the doses of 1.2 and 1.8 mg/kg/h and included flushing and inactivity.

### Long-tem infusion of dihydrocapsaicin in young cattle

**Figure 4:**
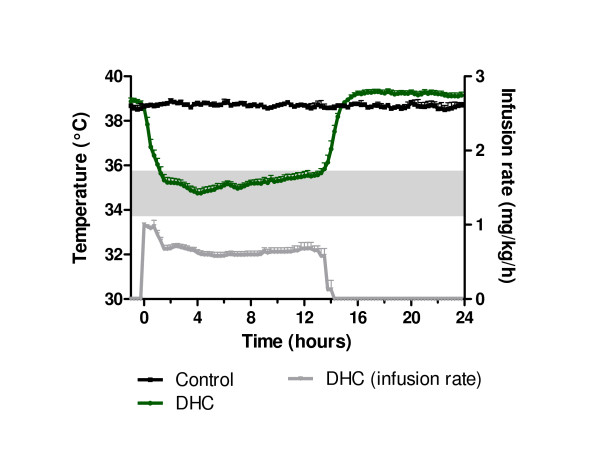
**Hypothermic effects of DHC infusion in monkeys**. Temperature profiles in conscious calves during intravenous infusion of various amounts of the transient receptor potential vanilloid type 1 agonist dihydrocapsaicin (n = 8) or control (n = 2). Target temperature of ΔT = -3 to -5°C are shown as shaded area. Statistics: Blood temperature was compared by a 2-way ANOVA followed by Bonferroni's post-test: ***p < 0.001 at t = 0.5 to 14 hours. Values are expressed as average ± SE, see Methods for further details.

Figure 4 summarizes infusion rate as well as the body temperature measured from t = -1 to 24 hours in calves treated with DHC or vehicle control. When young cattle were treated with DHC we observed an immediate and statistically significant decrease in body temperature from a baseline of approximately 39°C reaching the target ΔT of -3 to -5°C (shaded area) at t = 1.25 hour (***p < 0.01). The maximal ΔT of -4°C compared to vehicle control was observed after 4 hours infusion and the temperature remained at target until t = 13 hours in calves treated with DHC. When the infusion of DHC was stopped, the body temperature increased rapidly and to a level at approximately 0.5°C higher than that of control, though this was not statistically significant (p = ns). Clinical observations included panting, salvation/lacrimation, flushing and apparent discomfort and were evident especially in the first hour of the infusion were the dose administered was at 1.0 mg/kg/h, after this initial phase all clinical observations became less pronounced.

## Discussion

In the present study we demonstrate that continuous infusion of DHC, a TRPV1 receptor agonist, can induce a sustainable and clinically relevant mild hypothermia in rats, cynomologus monkeys, and in young cattle. In addition we show that also synthetic TRPV1 agonists such as MSK-195, olvanil, arvanil or rinvanil are able to produce hypothermia in rats. Similar, TRPV1 antagonists have been shown to induce hyperthermia in rats as well as in humans [28], which collectively demonstrate the central role of TRPV1 channels in the regulation of body temperature [11].

Based on death certificates, sudden cardiac arrest accounts for about 15% of all death in Western countries [29] and about 330,000 per year in the United States alone [30]. With the witness of a cardiac arrest and performance of CPR with return of spontaneous circulation (ROSC), mild therapeutic hypothermia has been shown to improve survival and neurological outcome [2]. At present the only therapy available is mechanical/physical cooling, which is associated with side-effects including pneumonia, sepsis, and stress-related shivering [7]. Moreover, it can be argued that mechanical cooling is connected to various practical issues that may affect the frequency of which the treatment is actually applied. Finally, the on-set of the use of mechanical cooling may take several hours following the ischemic insult [1-3], which in turn may affect the outcome of the treatment. Taken together there is an un-met medical need for the development of drug-induced mild therapeutic hypothermia, which may lead to a safer, manageable, and instant treatment immediately following ROSC. In animals drug-induced hypothermia has previously been demonstrated by activation of a number of biological systems located in the brain including the CB1 receptor [31,32], neurotensin receptor [33], muscarine receptor [34], and 5-HT-1A receptor [35]. Here we focused our efforts on the TRPV1 receptor agonists and investigated the feasibility of a long-term infusion-based method for chemically induced hypothermia.

Previously it has been shown that single administration of capsaicin, DHC, and RTX [25,26] can induce a short period of hypothermia in rats. In addition to the role in thermoregulation, the TRPV1 receptor is important in the sensation of pain and it contributes to the detection of noxious heat, which in turn may lead to effects on the cardiovascular and respiratory systems [36]. In the current study focused on temperature regulation, we in general did not observe any major behavioral changes that may indicate any of these effects, except in calves during the first 1 to 2 hours of the infusion, where some discomfort was observed indicating a pain reaction. Our findings may be explained by relatively low dose levels tested, or the fact that DHC was administered by the intravenous route, which could cause a relative distribution of DHC to central receptors possibly less involved in nociception. On the other hand, intravenous infusion of the TRPV1 agonist capsaicin was previously shown to cause a hypertension and tachycardia in anesthetized dogs [37]. Since the possible effect of DHC on the cardiovascular and system is central in understanding the therapeutic potential of the compound, the issue is currently a subject for specific studies. Notably, preliminary results demonstrate a dose-dependent cardiovascular effect including tachycardia and hypertension by continuous intravenous infusion of DHC [38]. Also, future studies may address possible adverse effects of infusion of DHC on coagulopathy, hematological effects, liver enzymes, and fluid and electrolyte shifts.

Besides the ability of inducing hypothermia *per se *to the relevant therapeutic level of 32-34°C, it is pivotal for a novel drug for the treatment of patients following ROSC after cardiac arrest, that mild therapeutic hypothermia can be sustained for an appropriate period of time. Thus, the current recommendation from ERC/AHA concerning the length of period of therapeutic hypothermia is 12-24 hours. Now, exposure of a TRPV1 agonist to the receptor may cause desensitization of the receptor [27], and accordingly repeated dosing may lead to a less pronounced hypothermic response as observed in young non-naïve animals [39]. In fact, very high exposure of a TRPV1 agonist may even lead to nerve fiber degeneration and subsequent re-innervation [40]. Here we investigated the possibility of sustaining hypothermia by applying various TRPV1 agonists by a continuous intravenous infusion to adult rats. Interestingly, our data indicate that the role of desensitization vary among the heterogeneous class of TRPV1 agonists, as it has previously been indicated *in vitro *in patch clamp studies [27]. Thus, infusion of arvanil caused a rapid desensitization towards the hypothermic effect, which was also seen at lower dose of arvanil (data not shown), whereas infusion of olvanil or DHC did not. Interestingly, olvanil caused a prolonged hypothermia even after the infusion was stopped, whereas the hypothermic effect of DHC was short-lasting after stopping the infusion. It is currently not known what causes this differential desensitization effect, but it may relate to the binding of the agonist to the receptor and/or the associated permeability of the ion channel [19,41]. Another possibility is that the observed difference in the pharmacological response of the various TRPV1 agonists is caused by different pharmacokinetic properties of the tested TRPV1 agonists.

From our initial screening of hypothermic properties DHC displayed a desirable profile showing a relevant and sustainable level of hypothermia as well as a rapid and controllable on- and off-set, and this candidate was therefore selected for further dose-response testing. An important component in the regulation of human body temperature is the ability to sweat, which causes heat loss via evaporative cooling. In contrast, in most animals including rats sweating does not contribute significantly to the body temperature homeostasis, but other mechanisms, such as panting, are important [42]. Therefore we included dose-response studies in rats as well as in cynomologus monkeys, the latter animal which in contrast to rats possesses the ability to sweat [43]. We observed that infusion of DHC caused a dose-dependent decrease in temperature in both rats and cynomologus monkeys. Notably, while the maximal response was at the same level, the sensitivity towards DHC was about 3-fold higher in the rats than in cynomologus monkeys. The reason for this is not clear, but may relate to the role of desensitization [39], since the cynomologus monkeys were dosed on repeated occasions, whereas the rats were all naïve to DHC. It may also be explained by species differences in the molecular structure of the TRPV1 receptor [19] or the components of thermoregulation [42,43]. Finally, it may play a role that DHC was formulated in different vehicles in the two experiments, which may have resulted in a difference in the exposure of the compound. In this context it is of interest that when the cynomologus monkeys, yet naïve to DHC, were treated for the first time at the dose of 0.3 mg/kg/h, the response was still less pronounced than rats treated with 0.25 mg/kg/h. This may indicate that desensitization is not the only reason for the observed difference in the hypothermic response.

Both rats and monkeys are small animals compared to humans. In order to test the hypothermic properties of DHC in a large animal at the weight of a human being, we infused DHC to young cattle. We were able to sustain hypothermia at the relevant therapeutic level for more than 12 hours. It should be noted that despite a constant infusion of compound, there was a minor incline in body temperature during that last hours of the infusion. Also, it is important to notice that we saw a minor overshoot in the body temperature after discontinuation of the infusion. Likewise a minor overshooting was observed in the cynomologus monkeys following the highest dose (1.8 mg/kg/h) applied. The reason for this overshooting is not clear at present, and it is not known whether or not it may be prevented by a gradual discontinuation of the infusion or by the treatment of the fever with non-steroidal anti-inflammatory drugs or similar. Also, due to technical limitations here we report only peripheral body temperatures, whereas the brain temperature, was not assessed. These matters should be subject of further experimentation.

In rat models of ischemia a beneficial effect of mild hypothermia on neurological outcome has been demonstrated by infusion of the neurotensin receptor agonist NT77 [44] or a cannabinod (CB) receptor agonist WIN 55,212-2 [45]. However, the use of a CB1 agonist for inducing hypothermia may be connected with undesirable cardiac and respiratory side-effects [46,47]. In the present study we demonstrate for the first time that a sustainable mild therapeutic hypothermia relevant for the human situation can be obtained in rodents and small and large non-rodents. Interestingly, feasibility studies in a rat model of cardiopulmonary resuscitation have demonstrated that infusion of DHC can also induce mild hypothermia in this model, and in a similar fashion to the one reported here [38].

## Conclusions

Taken together our data support the hypothesis that infusion of dihydrocapsaicin is a candidate for testing as a primary or adjunct method of inducing and maintaining therapeutic hypothermia.

## Competing interests

The study was sponsored by Neurokey AS. At the time of the study K. Fosgerau, M Jayatissa, JW Gotfredsen, UJ Weber and C Videbaek where employed at the company. JW Gotfredsen, UJ Weber, L Køber and C Torp-Pedersen were founders of the company.

## Authors' contributions

KF, UW, JG, MJ, CB, NK, MV, PT, AS, PH, JR: have made substantial contributions to conception and design, or acquisition of data, or analysis and interpretation of data, and writing of article. LK, CT, CV: have been involved in drafting the manuscript or revising it critically for important intellectual content. All authors have read and approved the final manuscript.

## Pre-publication history

The pre-publication history for this paper can be accessed here:

http://www.biomedcentral.com/1471-2261/10/51/prepub
